# Intersection of policy and Immunization Information Systems (IIS)

**DOI:** 10.1186/s12889-023-16457-2

**Published:** 2023-09-20

**Authors:** Lara A. Heersema, Luke Cunniff, Amanda L. Eiden, Isha Sharma, Jaya Mishra, Alexandra Bhatti

**Affiliations:** 1 Merck & Co., Inc, 351 North Sumneytown Pike, North Wales, PA 19454 USA; 2 Medicine and Health Behavior, University of North Carolina at Chapel Hill, Chapel Hill, NC USA

**Keywords:** Immunization information systems, Vaccines, Public health, Children’s health, Health information systems, Health information exchange, Health information technology, Health policy, Legal study

## Abstract

**Background:**

Immunization information systems (IIS) are confidential, population-based computerized databases that record vaccination doses administered to persons residing within a given geopolitical area. We sought to highlight the evolution of IIS policy over the last two decades, as IIS play a pivotal role in achieving equitable and high vaccine uptake.

**Methods:**

Legal epidemiological research methods were used to assess relevant IIS statutes and administrative codes across all 50 states, the District of Columbia, Philadelphia, and New York City. Where relevant, laws were cross-checked or supplemented with state and local health department resources. Comparisons to previous legal studies enabled evaluation of trends in IIS laws over time.

**Results:**

The compilation of current laws provides an updated overview of the diverse interstate and intrastate policies within the US that govern the capabilities and implementation of IIS. The findings of this study show the progress that has been made in the past decade in improving policies that enable IIS to be utilized across the life-course. Conversely, gaps in IIS data collection, limited interoperability with local and national health information systems, and inconsistent access to view or utilize IIS records due to existing policies, continue to limit the full potential of IIS.

**Conclusions:**

In the United States (US), IIS are implemented and managed at the state and local level, creating variability in IIS policies and implementation. Findings from this study serve as a comprehensive benchmark of current IIS laws that may aid policy stakeholders who are exploring amendments to jurisdictional IIS laws.

**Supplementary Information:**

The online version contains supplementary material available at 10.1186/s12889-023-16457-2.

## Background

Immunization information systems (IIS) are confidential, population-based, computerized databases that record, consolidate, and report vaccinations administered by participating providers to persons residing within a given geopolitical area [1]. The COVID-19 pandemic has underscored the pivotal role that IIS (also known as vaccine registries) play in supporting vaccination uptake, increasing vaccination rates, and therefore, disease prevention. At the point of clinical care, a well-integrated IIS can improve identification of an individual’s vaccination needs and facilitate data-sharing across health systems. At the population health level, IIS can be used to monitor vaccination rates within communities, better target interventions to support vaccine uptake, and mitigate vaccination disparities [2].

Currently, in the US, IIS are operated at the jurisdictional level with no national centralized repository for vaccination data. There are over 60 different state, tribal, city, and territorial IIS [3]. Disparate IIS operations and legal frameworks can translate into variability in technical and programmatic capacity [4, 5]. While progress has been made in strengthening and expanding IIS, further action is needed to help realize the full value IIS can provide in supporting vaccination across the life-course [6]. A critical component needed to identify opportunities for strengthening IIS is understanding the current legal landscape of IIS laws across the country [5].

A comprehensive legal assessment of IIS laws has not been completed since 2012 [7]. The objective of this study was to establish an updated database of jurisdictional laws related to IIS and their ability to receive, store, or disclose vaccination information across the life-course. Jurisdictional law trends were also compared over the past few decades to identify opportunities for strengthening IIS policies to improve public health outcomes.

## Methods

### Study design

We used legal epidemiological research methods [8] to complete a cross-sectional analysis of IIS laws. This included a legal search of relevant statutes and regulations to develop a database of IIS laws across all 50 states, the District of Columbia (DC), Philadelphia (PHI), and New York City (NYC). The final study population included 53 jurisdictions that were included in the CDC’s online registry list at the time of analysis [9]. This research was supported through search criteria coded within a total of 19 questions to better understand the nuances of the law. Variables for analysis were selected for comparison with prior studies (Table 2) and the study intent to understand the impact of IIS policies across the life-course. We used a four-stage process to establish the coding questions used to analyze specific variables in state laws: (1) development of a question set; (2) testing questions on a batch of legal provisions; (3) analysis of question adequacy; and (4) revision of questions. Final questions established for coding included: (i) what ages were included in the IIS; (ii) whether and which entities were required to report pediatric or adult vaccine administration to the IIS; (iii) whether there was a mechanism in place for enforcing reporting; (iv) what type of consent was required from a parent or guardian to include children’s data; (v) whether the IIS law permitted intrastate and interstate data sharing provisions; (vi) whether IIS may be used for school and childcare entry; (vii) whether schools and childcare facilities had read-write access to the data; and (viii) whether inclusion of certain demographic data elements were required. A full list of the final coding questions and definitions is available in the online supplemental materials (Additional file 1: Exhibit A1) [10].

Active state laws were collected between July 5th and July 16th, 2021, using the WestlawNext^®^ legal database and underwent a series of validations with independent coders reviewing each coding decision from August 2021 to February 2022.Each jurisdiction’s laws were divided among five independent coders for analysis and coded against the legal variables. A minimum of two independent reviewers conducted an evaluation and validation of the final dataset values with the support of state sources such as jurisdiction health department websites. Independent evaluators reviewed all coding decisions to ensure accuracy and quality. Discrepancies were identified and aligned upon by a minimum of three reviewers for final coding decisions. We also identified whether there were ambiguities in the law which precluded coding decisions. Across our coding questions, if the law was silent on a particular coding attribute, we coded it to indicate that the attribute was not mentioned.

### Analysis

Analyses of legal data were conducted from August 2021 to February 2022. Data was collated using Microsoft Excel and geographical maps were produced using JMP (version 15.2.0). See online supplemental materials for a complete jurisdiction-based coding table (Additional file 1: Exhibit A1) [10].

Changes in IIS laws were also assessed over time utilizing previously published legal assessments and surveys of immunization program managers conducted in 1995 [11], 1997–2000 [12], 2010–2011 [13], and 2011–2012 [7] (Table 2).

## Results

See Additional file 1 for comprehensive coding results and jurisdiction examples of legal text [10]. See Additional file 1: Exhibit A2 for WestlawNext^®^ legal database pincite location at the time of collection [10].

### Ages included in the IIS

Laws from all 53 jurisdictions assessed, except for CT, reference vaccination data collection within the IIS for all ages. Under CT law, “‘[i]mmunization registry’ means the department’s ongoing computer-based registry of children who have not yet begun first grade of school and their complete immunization history” [10]. While CT law expressly anchors to “children” when describing ages included in the IIS, the state website was more ambiguous and references that patients may access their immunization record from the state IIS – and did not specify that it was childhood only [14]. Because the official website was ambiguous, coders ultimately coded CT law as a childhood registry, based on the legal text. Even though the remaining 52 jurisdictions (98%) had the capacity to retain vaccination data for all ages, there were nuances within the law that may impact the quality and completeness of data across the life-course.

### Vaccination reporting requirements

#### Pediatric reporting requirements

Forty-one states, DC, NYC, and PHI (83% of jurisdictions) required all or certain providers to report all pediatric vaccinations to the IIS (Table 1B). Of these states, under NJ law, providers were required to report vaccinations administered to certain age groups within the pediatric population to the IIS: “health care providers shall report to the NJIIS the administration of a vaccine to a child less than seven years of age within 30 days of administration” [10].

**Table 1:** Overview of findings for selection of coding questions used to assess IIS statutes and administrative codes across all 50 states, the District of Columbia, Philadelphia, and New York City

**Section A: Consent required for birth records (birth), pediatric vaccination records (ped), and adult vaccination records (adult) to be included in IIS**															
Consent	Mandated (no opt-out)			Implied (with opt-out)			Expressed (written)			Expressed (written or verbal)			Not mentioned		
Record	Birth	Ped	Adult	Birth	Ped	Adult	Birth	Ped	Adult	Birth	Ped	Adult	Birth	Ped	Adult
Jurisdictions Number (%)	14 (26)	15 (28)	9 (17)	7 (13)	28 (53)	29 (55)	2 (4)	2 (4)	4 (7)	1 (2)	1 (2)	3 (6)	29 (55)	7 (13)	8 (15)
**Section B: Provider types required to report vaccinations for children (child) and adults (adult)**															
Provider	At least 1 Provider type			All vaccine providers		Pharmacists only			Medicaid / Public / VFC only		Other^a^		Not mentioned		
Patient Age	Child	Adult		Child	Adult	Child	Adult		Child	Adult	Child	Adult	Child	Adult	
Jurisdictions Number (%)	44 (83)	38 (72)		30 (57)	20 (38)	6 (11)	7 (13)		0 (0)	1 (2)	8 (15)	10 (19)	9 (17)	15 (28)	
**Section C: School and/or childcare utilization of IIS**															
	Accept IIS as proof of vaccination record					At least 1 staff member has read access					At least 1 staff member has edit permission				
Utilization	Yes	No		Not mentioned		Yes	No		Not mentioned		Yes		No	Not mentioned	
Jurisdictions Number (%)	43 (81)	1 (2)		9 (17)		44 (83)	4 (7)		5 (10)		22 (41)		13 (25)	18 (34)	
**Section D: Mandatory demographic elements included in IIS**															
Demographic Element	Date of Birth			Sex			Race		Ethnicity				Birthplace		
Jurisdictions Number (%)	27 (51)			25 (47)			13 (25)		9 (17)				15 (28)		
**Section E: Patient allowed direct access to records through digital portal or application**															
Access	Yes						Adult records only						No		
Jurisdictions Number (%)	18 (34)						3 (6)						32^b^ (60)		

^a^Laws may require specific provider type(s) to report such as optometrists, student health clinics, dentists, midwives, Vaccines for Children (VFC), etc. depending on state and patient age

^b^As of publication, NJ allowed access to COVID-19 vaccination records only

There was variability in the types of providers required to report pediatric vaccinations to the IIS. Twenty-eight states, NYC, and PHI (57%) required all providers to report pediatric vaccinations to the IIS; in contrast, six jurisdictions (11%) only required pharmacists to report. The remaining eight jurisdictions (15%) required specific provider types to report pediatric vaccinations or had requirements for only select vaccines. For example, under CA law, pharmacists and optometrists must report vaccinations to the IIS [10]. On the other hand, per IL law, all active Vaccine for Children (VFC) providers and dentists are required to report to the IIS [10]. In some cases, the reporting requirements were also vaccine dependent. For instance, under OK law, “Licensed Midwives shall implement a procedure to ensure that the hepatitis B vaccination is administered to all live infants within twelve (12) hours of birth and recorded in the Oklahoma State Immunization Information System” [10].

#### Adult reporting requirements

Thirty-five states, DC, NYC, and PHI (72%) required some provider reporting of adult vaccination to the IIS. Nineteen states and PHI (38%) required all providers to report adult vaccinations to the IIS (Table 1B). Six states and DC (13%) required that only pharmacists report adult vaccination data to the IIS. The remaining 11 jurisdictions (21%) required that other specific provider types report adult vaccination data to the IIS. As an example of age-based reporting requirements, per AR law, “[a]ll Providers shall report to the Department the administration of any childhood immunization to any person under twenty-two years of age” [10]. Other states such as OR and RI required that any provider administering state-supplied vaccinations, such as those through a Universal Purchase program, must report vaccinations administered in the IIS [10, 15].

#### Mechanisms to enforce reporting requirements

Seven jurisdictions (13%) expressly included penalties to encourage compliance with provider reporting requirements. Penalties across these jurisdictions varied from monetary penalties and vaccine ordering restrictions to referral to the relevant licensing board or removal from the VFC program. For example, AR law stated that “[f]ailure to report shall result in the Department contacting the Provider to encourage compliance. Continued non-compliance may result in sanctions not to exceed $25.00 and/or removal from the Vaccine For Children (VFC) program” [10]. Under ME law, “[i]ntentional or knowing violation of any of these rules by an Immunization Provider may result in a request for disciplinary action by the appropriate licensing and regulatory board which has regulatory authority over the Immunization Provider”(see Additional file 1 component 2 for additional examples, and Additional file 1: Exhibit A2) [10].

### Patient consent required for data inclusion in IIS

#### Consent for inclusion of pediatric data in IIS

In 14 states and NYC (28%), pediatric data was included in the IIS and the law did not mention patient or parent/guardian ability to “opt-out” or remove their data from the IIS (Table 1A). For example, under OR law, “[a]ll children born in the state shall be enrolled in the IIS” [10]. In about half of states (27) and PHI (53%), consent for data inclusion was implied; however, the law specifically stated that patients or parent/guardians could “opt-out” from the IIS. Under MD law, “. . a health care provider who administers a vaccine. . shall. . Notify the individual or the parent or guardian of a minor of the right to refuse to disclose to ImmuNet” [10].

Three states (6%) required consent for pediatric data to be reported into the IIS, while in 7 states and DC (15%), the law was ambiguous or silent as it relates to consent. Written consent is required in MO and TX, whereas MT law did not specify that consent must be written. For example, under MO law, “[t]he patient shall attest to the inclusion of such information in the system by signing a form provided by the pharmacist” [10]. Unlike MO, MT law states that, “[a] pharmacist who administers an immunization pursuant to this section shall. . offer the patient the opportunity to have the immunization information reported to the state immunization information system” [10]. As written, MT law implies consent is required for inclusion in the IIS, as it provides the opportunity for a patient to have their data reported to the IIS, or “opt-in”, as opposed to implied consent where a patient data is included unless a patient “opts-out” (see Additional file 1 component 3 for more examples) [10].

#### Consent for inclusion of adult vaccination data in IIS

Regarding inclusion of adult vaccination data in the IIS, nine states’ laws (17%) did not specify that patients could “opt-out” or remove their data from the IIS; in eight states and DC (17%), the law was ambiguous or silent as it relates to consent (Table 1A). Like pediatric consent requirements, in 28 states and PHL (55%), consent was implied and per law, patients had the ability to “opt-out” or prevent data-sharing with the IIS. For example, UT law stated that, “[i]mmunization records may be included in the system unless the individual or guardian withdraws from the system. An individual or guardian may withdraw from the system at any time” [10]. Seven states (13%) required express consent for adult data to be included in the IIS. Four jurisdictions required written consent whereas three jurisdictions did not specify that consent must be written. Per NJ law, adults may opt-in, or register to have their data included in the IIS, “[a]n adult registrant may enroll in the NJIIS” [10]. Florida law was more nuanced, since consent was implied with opt-out for certain adult age groups, but express consent was required for college students who were not between 18 and 23 years of age (see Additional file 1 component 3 for more examples) [10].

### Data sharing provisions (intrastate and interstate)

#### Intrastate data-sharing permissions

Thirty-nine states and NYC (75%), expressly reference intrastate data sharing. This is data sharing that includes an exchange of data between the IIS and other data systems within the state and may specify entities, persons, or reasons for information sharing. For example, WV law provided a detailed list of permitted disclosures including how the data should be used and by which entities, such as local health departments, licensed providers, and school officials, among others (see Additional file 1 component 4 for more examples) [10]. Other states provided broader data sharing provisions. For example, under MI law, “. . the department may transmit transcripts or copies of public health records or reports to state or national secure public health data systems or individuals responsible for the health care of a person if the records or reports relate to residents of other states or countries. . .” [10]. In this case, precisely what entities may access the data were not specified, compared to WV.

#### Interstate data-sharing permissions

Only 22 (42%) states’ laws expressly identified interstate data-sharing permissions, or data exchange across state lines. States may share data across states through broader public health data sharing provisions (similar to intrastate provisions), which may not be captured in this analysis if the laws did not expressly reference IIS data-sharing. Per ME law, “[t]he Department may exchange information with other immunization registries and/or immunization databases maintained by health maintenance organizations and health insurance companies” [10]. Similarly, under MA law, “[t]he department may enter into collaborative agreements with registries of other states and exchange individual or group information provided that maximum protections are afforded the confidentiality of citizens of the commonwealth in accordance with state law” [10].

### School and childcare use of IIS

#### Proof of vaccination for admission

Forty-one states, DC, and NYC (81%) expressly stated that the IIS or an official copy of records obtained from the IIS was valid proof of vaccination for school or childcare entry (Table 1C). For example, CO law stated, “An electronic file or hard copy of an electronic file provided to the school directly from the immunization information system” was considered an official school record [10]. At least fifteen states defined specific aspects of the record that must be present for school or childcare admission. For example, AR law qualified that a printed record “of the statewide immunization registry with the Official Seal of the State of Arkansas is an approved immunization record” [10].

#### School and childcare access to IIS

**Fig. 1:**
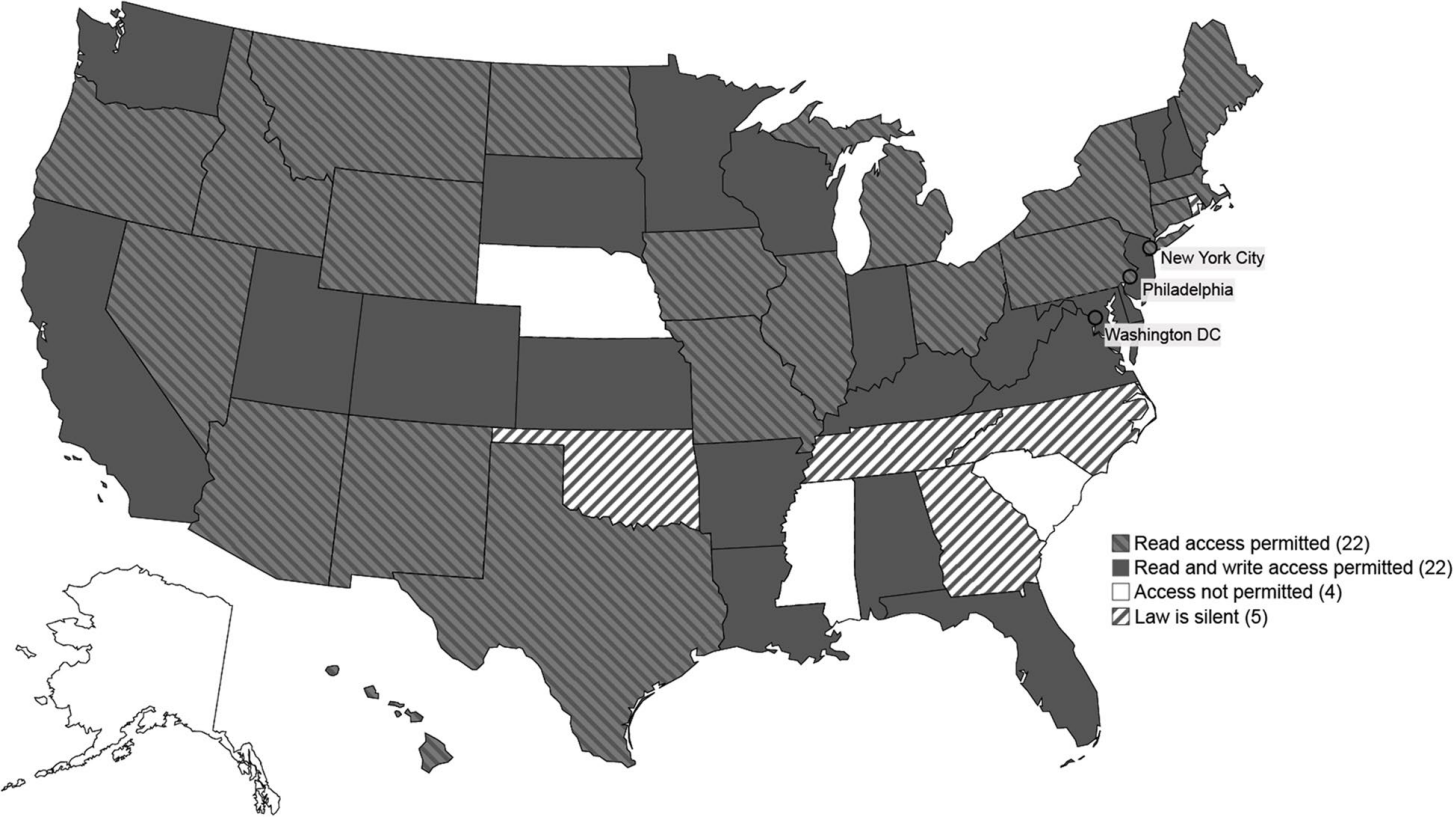
School and childcare read and write/edit access to IIS. Notes: Read access permitted indicates that at least 1 school or childcare staff member is permitted access to view records in the IIS. Read and write access permitted indicates that at least 1 school or childcare staff member is permitted access to view and or edit records in the IIS. Access not permitted indicates that provisions in the law prohibit or omit school and childcare staff members from direct IIS access. Law is silent indicates that school or childcare access to IIS records was not addressed in the law

Forty-four (83%) jurisdictions allowed school or childcare personnel direct access to the IIS, meaning that personnel are users of the IIS system and are at least able to view records Table 1C, Figure 1). Of these jurisdictions, 11 explicitly limited IIS access to children under their care. For example, NJ law limited access such that “[c]hild care centers, schools, colleges, and universities shall only access immunization information on a registrant that they have enrolled or are in the process of enrolling into their institutions” [10]. Oregon law also included a provision that could require an authorized user to, “provide evidence that such client was under the care of the person or enrolled in the person’s post-secondary educational institution, school, children’s facility, program or health plan at the time the client’s record was accessed” [10].

Twenty-two (42%) jurisdictions allowed write/edit access of IIS records to school and childcare personnel (Table 1C). For example, SD law stated that if a card is presented as proof of vaccination for children entering childcare programs or schools, then “the immunizations shall be entered in to SDIIS. . .” [10]. Some jurisdictions with write/edit access have additional restrictions on which facilities or personnel have write/edit access; for example, AR law included “school nurse or other health official who has direct or supervisory responsibility for the delivery of immunizations falls within the definition of ’health care professional’” [10] but excluded other school or childcare personnel (see Additional file 1 component 5) [10].

### Inclusion of demographic data (e.g., race/ethnicity)

**Fig. 2:**
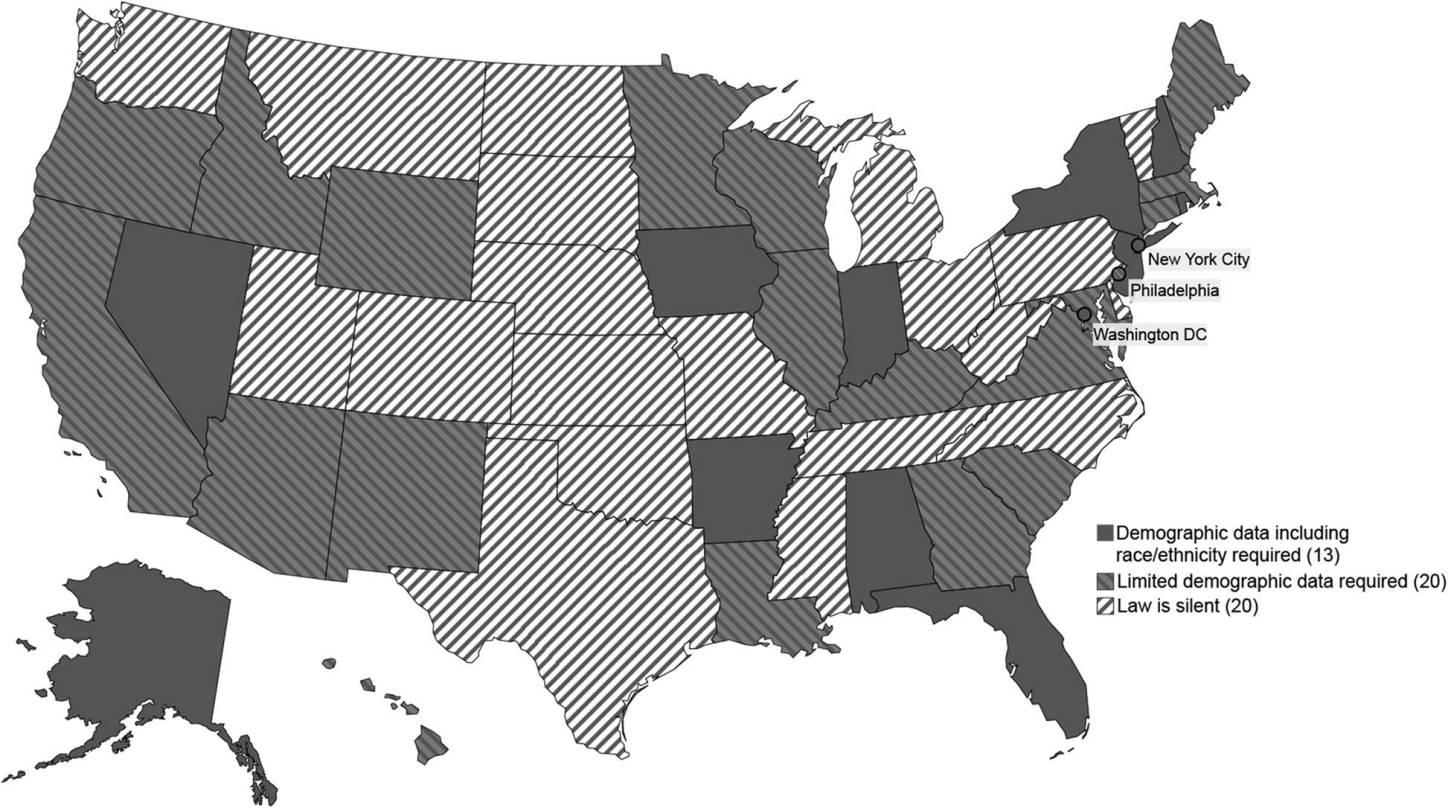
IIS demographic data reporting requirements. Notes: Limited demographic data indicates that demographic data was mentioned in the law but may not have specified which aspects of demographic data is required for reporting or may be limited to date of birth and address. Jurisdictions that specified reporting of additional demographic data, specifically race and/or ethnicity, are considered separately. Law is silent indicates that collection or reporting of demographic data was not addressed in the law

Jurisdictional law may dictate that certain patient demographic data are required elements to report into the IIS; although, jurisdictions may collect additional demographic data beyond what is expressly required by law. Thirty states, DC, NYC, and PHI (62%) expressly identified certain required demographic data that must be included in the IIS (Table 1D). There was great variability in the data elements required across these jurisdictions. For example, 22 states, DC, NYC, and PHI (47%) specified that sex is a required data element; however, only 11 states, DC, and NYC (25%) required race data and seven states, DC, and NYC (17%) required ethnicity data (Fig. 2).

Twenty-three states, DC, NYC, and PHI (49%) expressly required a current address as a data element in the IIS. Under NJ law, “[h]ealth care providers shall report. . the following required data fields. . : 1. Complete name; 2. Date of birth; 3. Ethnicity/Race; 4. Gender; 5. Address. . .” [10]. This is compared to states like HI, which specified minimal demographic data, “(b) After the vaccination is administered, the pharmacist shall immediately provide to the patient a vaccination record including the following information:. . The patient’s name and date of birth. . .(c) the pharmacist shall provide…the department of health immunization registry the same information provided to the patient pursuant to subsection (b). . .” [10].

### Direct patient access to vaccination data through the IIS

Sixteen states, DC, and NYC (34%) allowed patients or a patient’s parents or guardians direct access to view their records from the IIS through an online portal or mobile application (Table 1E). Three additional states (6%) allowed direct access to adult patient records only. Illinois also allowed parents or guardians to request their child’s record through an online form. The remaining jurisdictions (60%) did not provide direct online access to vaccination records for patients. Beginning in 2021, NJ allowed patients to access only their COVID-19 vaccination records using a mobile app [16].

## Discussion

This was the first comprehensive legal assessment of IIS laws within the US since 2012, to the authors’ knowledge [7]. This study of IIS laws across the US primarily examined multiple attributes of the laws and nuances within each jurisdiction. Previous studies focused primarily on pediatric vaccination, the legal authorization of an IIS, privacy and type of consent required for collecting patient information, provider reporting requirements, and laws addressing information sharing (Table 2). Our study expanded upon previous research by including school and childcare related IIS provisions, demographic-related provisions, and patient/parent direct access to vaccination data (Table 1). Additionally, this study built on previous analyses to identify trends that may support or constrain the ability of an IIS to receive, consolidate, or disclose vaccination information across the life-course (Table 2). The impact of IIS laws and their changes over time are discussed in the following sections in the context of IIS users and public health outcomes. Decision-makers can leverage these findings to understand the range of options when exploring IIS policy development.

**Table 2:** Comparison of previous and current legal epidemiology studies of immunization information systems and immunization registries across the United States

**Section A: Sources of information & population age vaccination records studied**															
Study	Current Study (2022)			Martin et al. (2015)			Hedden et al. (2012)			Horlick et al. (2001)			Gostin and Lazzarini (1995)		
Primary Source	WestlawNext^®^ legal database, July 2021			WestLaw legal database, Oct-Nov 2011			Legal databases, 2010-2011			Survey and Phone Interviews, 1997-1998, 2000			Not reported		
Secondary Sources	Public health department websites			Survey of immunization program managers. Phone interviews^a^			Library records of statues and regulations			Legislation, administrative rules, and immunization registry policies			Not reported		
Focus population	Pediatric and Adult			Pediatric and Adult			Pediatric			Pediatric			Pediatric		
Jurisdictions included	53			53^b^			56^c^			51^d^			52^e^		
**Section B: Change in ages included in IIS**															
Reporting ages	No age restrictions (all ages)							Pediatric only (0-18)							
Study	2022	2015		2012	2001	1995		2022		2015	2012		2001	1995	
Jurisdictions Number (%)	52 (98)	51 (96)		-	-	-		1 (2)		2 (4)	47 (84)		36 (71)	16 (31)	
**Section C: Change in consent required for pediatric vaccination reporting**															
Consent	Mandated (no opt-out)			Implied (with opt-out)			Expressed (written)			Expressed (written or verbal)			Not mentioned		
Study	2022	2015	2001	2022	2015	2001	2022	2015	2001	2022	2015	2001	2022	2015	2001
Jurisdictions Number (%)	15 (28)	12 (23)	12 (24)	27 (51)	38 (72)	23 (44)	2 (4)	2 (4)	12 (24)	1 (2)	1 (2)	2 (4)	8 (15)	-	2 (4)
**Section D: Change in mandated pediatric vaccination reporting required for at least 1 provider**															
Study	2022			2015		2012				2001			1995^f^		
Jurisdictions Number (%)	44 (83)			31 (58)		26 (46)				12 (24)			12 (63)		
**Section E: Change in age restrictions for mandated vaccination reporting by at least 1 provider**															
Reporting age restrictions	No age restrictions (all ages)				Adult subset^g^			Pediatric (all under 19)				Pediatric subset^g^			
Study	2022		2015		2022		2015	2022		2015		2022		2015	
Jurisdictions Number (%)	36 (68)		14 (26)		2 (4)		3 (6)	6 (11)		12 (23)		2 (4)		2 (4)	

^a^Phone interviews conducted with relevant individuals including immunization program and IIS managers, CDC public health advisors, etc

^b^Compared to current study - Excludes NH. Includes San Antonio, TX

^c^Compared to current study - Includes San Antonio and Houston, TX and Chicago, IL

^d^Compared to current study - Excludes NYC and PHI

^e^Compared to current study - Excludes NYC and PHI. Includes Puerto Rico

^f^Mandated reporting to immunization registry. An additional 11 states required reporting to local health department

^g^Subset of adult and pediatric age groups indicate that some jurisdictions require reporting for only a portion of the age group

The CDC has identified three broad user types and corresponding functions for IIS records: (1) patients and/or the patient’s guardian use of IIS information to determine their vaccination needs and to show proof of vaccination; (2) health care providers use of IIS at the point of clinical care to determine appropriate vaccinations during a patient visit; and (3) public health officials use of IIS to get aggregate population-level data for surveillance and program development [17]. The findings show that while progress has been made in updating policies to support these areas in the jurisdictions studied, gaps in data collected or access to IIS remain, limiting the full potential of IIS. For example, with collecting complete population level data, almost all 53 jurisdictions assessed permit IIS record collection across the life-course, however, only 42% of jurisdictions expressly referenced that schools or childcare centers have edit rights within an IIS (Table 1C). This could reduce data completeness in an IIS since school and/or childcare staff often have the most complete vaccination data for students due to requirements for enrollment [18].

Another important gap identified was direct access for patients to view their vaccination records in the IIS, which can empower patients or patient guardians to make informed decisions about their health. Direct access to an IIS also offers patients the ability to provide proof of required vaccination, without requesting them through a vaccination provider or public health professional. Despite the value provided by allowing for patient access, only 34% of jurisdictions provide patients direct access to view their own vaccination data from the IIS (Table 1E).

Additionally, increasing IIS interoperability with Electronic Health Record (EHR) systems and intrastate and interstate data sharing can help improve surveillance and reduce data quality challenges [19]. Before three years of age, over 20% of children in the US have typically seen more than one health care provider resulting in scattered medical records, underscoring the importance of enabling the secure exchange of vaccination data [20]. While, jurisdictions with laws that specifically allowed for intrastate data-sharing have increased from 22 to 36 since 2012, only 38% of jurisdictions expressly allow for interstate data sharing, potentially hindering transfer of important vaccination records for many patients across states lines. This is especially critical given the movement of individuals and families between states. According to the 2018 American Community Survey, an estimated 31% of native US residents were born in another state, leaving individuals with the challenge of tracking their vaccination records across the life course [21].

Immunization information systems can also have a direct impact on vaccinations at the point of care by notifying patients that they are due for vaccinations and by supporting providers in determining appropriate vaccinations; preventing both over- and under- vaccination [22, 23]. IIS policies may impact whether a vaccine provider uses an IIS to report vaccinations or review vaccination history [24, 25]. Today, most jurisdictions expressly identified at least one provider type required to report pediatric and adolescent vaccinations (83%) as well as adult vaccinations (72%). On the other hand, all vaccination providers were required to report in only 57% and 38% of jurisdictions for pediatric and adolescent, and adult patients, respectively (Table 1B). While the number of jurisdictions requiring at least one provider type to report pediatric and adult vaccinations has increased over the past decade (Table 2D-E), requiring all vaccine providers to report could help reduce gaps in patient records and care [23].

Furthermore, IIS records guide public health action. Public health officials and researchers use IIS to understand vaccination coverage, examine vaccination disparities, develop targeted vaccination efforts, and assess vaccination efficacy when vaccine-preventable diseases are present in a community [22]. The CDC has designed functional standards that describe what is needed by an IIS to best support immunization programs, providers, and other stakeholders. This infrastructure includes data elements like race, ethnicity, address, birthplace, gender, insurance status, and VFC eligibility. Collecting these demographic characteristics is a critical component of understanding a population and addressing vaccination disparities amongst population groups. We found only 25% of jurisdictions’ laws detail race or ethnicity as mandatory patient data for inclusion in the IIS (Table 1D). Therefore, many vaccination programs are currently limited by the type of data that can be used to both understand what disparities may exist in their community as well as inform their outreach strategies.

The COVID-19 pandemic has exposed gaps in current vaccination data systems but has also created opportunities to modernize IIS infrastructure and legal frameworks, given the increased attention from policymakers and additional federal funding. Privacy, consent, and access continue to be consistent themes in IIS policy, especially in the context of the COVID-19 pandemic. These considerations must be weighed along with the essential need for IIS that are able to manage patient records efficiently and effectively and securely exchange information among states, across providers, and public health agencies to support vaccination efforts. Policymakers and public health stakeholders can also leverage behavioral economics and experience design when updating IIS laws and systems to increase participation and the inclusion of the broader population (e.g., leaning into “opt-out” IIS inclusion policies via implied consent as the status quo for collecting patient data, unless a patient “opts-out”).

### Limitations

This study was a comprehensive legal assessment of IIS laws with supplementary data from state health department websites. It did not include surveys or interviews of relevant jurisdictions operating an IIS. There may be programmatic policies in places that impose additional requirements that have not been included in this analysis. Local rules, policies, and municipal ordinances were not used unless incorporated within jurisdiction’s law or health department websites. Given rapidly evolving policy landscape due to the COVID-19 pandemic, some laws may have changed since the date of data collection.

## Conclusions

Immunization information systems are critical tools that support a robust and resilient vaccine ecosystem and broader health system infrastructure [2]. They serve as a centralized repository of vaccination data across the life-course with the capacity to support emergency preparedness and pandemic planning, integrate with other electronic health information systems, identify patient needs, and support addressing vaccination gaps and disparities at the population health level [2]. Findings from this study highlight the evolution of IIS policies over the last two decades, serve as a comprehensive benchmark for future analyses, and may help policy stakeholders who are exploring amendments to jurisdictional IIS laws. Future research should explore the impact of these variable laws on the operationalization of IIS and vaccination coverage rates, as well as how they may support or hinder programmatic efforts to improve vaccination coverage rates and address vaccination disparities within communities.

### Supplementary Information

**Additional file 1.**

## Data Availability

The datasets generated during and/or analyzed during the current study are available from the corresponding author on reasonable request.

## Abbreviations

DC: District of Columbia
EHR: Electronic Health Record
IIS: Immunization information systems
NYC: New York City
PHI: Philadelphia
US: United States
VFC: Vaccine For Children
