# Supplementary material for: Intersection of policy and Immunization Information Systems (IIS)
Source: BMC Public Health. 2023 Sep 20;23:1828. doi: 10.1186/s12889-023-16457-2 (PMC10510248; doi:10.1186/s12889-023-16457-2)
Supplement: Supplementary file 1 — Additional file 1. [file 12889_2023_16457_MOESM1_ESM.docx]

TITLE

Intersection of Policy and Immunization Information Systems (IIS)

AuthorS

Dr. Lara A. Heersema, PhD^1^

Mr. Luke Cunniff, MIA^1^

Dr. Amanda L. Eiden, PhD, MBA, MPH ^1^

Ms. Isha Sharma, MPH^2^

Ms. Jaya Mishra^3^

Ms. Alexandra Bhatti, JD, MPH^1^

^1^Merck & Co., Inc., Rahway, NJ, USA

^2^former employee of Merck & Co., Inc., Rahway, NJ, USA

^3^University of North Carolina at Chapel Hill, Medicine and Health Behavior, Chapel Hill, NC, USA former intern of Merck & Co., Inc., Rahway, NJ, USA

Corresponding Author

Name: Luke Cunniff

Mailing address: 351 North Sumneytown Pike, North Wales, PA 19454, USA

Email address: luke.cunniff@merck.com

Appendix

**Supplementary Materials:** *Extended methods, examples, and details regarding all 50 states, District of Columbia (DC), Philadelphia (PHI), and New York City (NYC) immunization information system (IIS) laws*

**Component 1. Methods - continued**

First, we examined which ages were included in the IIS. We organized states by either “all ages” or “children only (0-18 years).” Second, we examined whether any entities were required to report pediatric vaccinations to the IIS, and if so, which entities. We coded pediatric reporting requirements as “all providers”, “pharmacists only”, or “other”. “Other” was defined as any other provider type(s) that were required to report pediatric vaccinations. For example, a jurisdiction was coded “other” if it required both pharmacists and dentists to report pediatric vaccinations. Third, we examined provider requirements for adult vaccinations, which took the same approach as pediatric reporting requirements. We then examined which enforcement mechanisms were in place to ensure compliance with reporting requirements.

Next, we assessed the type of consent required from a parent before Vital Statistics shares birth record information of their child with an IIS. Additionally, we assessed the type of consent required from a parent before reporting vaccination information for their child to an IIS. Consent required from an adult before vaccination information is reported in an IIS was also recorded. Coding categories included: expressed consent (either verbal or written), expressed consent (written only), implied consent with opt out, or mandated reporting with no opt out. Laws that did not mention, or did not clearly mention, consent of minors was coded under “not mentioned” or “unclear.”

We also assessed intrastate and interstate data-sharing provisions. Intrastate data sharing provisions were defined as legislation that referenced the disclosure or transfer of data within the IIS to other entities within a state. Similarly, interstate data sharing provisions were defined as legislation that references the disclosure or transfer of IIS data to other states, including other states’ IIS. We additionally identified whether the intrastate or interstate data sharing provisions also contained requirements that prescribed how, and under what conditions, that data may be shared.

Additionally, we identified whether state laws expressly identified IIS records or certificates as acceptable proof of vaccination for school or childcare facility entry. We further assessed whether immunization records could be disclosed to school or childcare facilities; whether school or childcare facility staff had read access to the IIS, through which they could review data only; and whether schools or childcare facilities have expressed edit rights to update or add data to the IIS as authorized users.

Finally, we assessed whether inclusion of demographic data is required within the IIS. If the state required inclusion of demographic data, we included the associated language used to characterize the details of the demographic data and the specific elements of demographic data required to be included in the IIS (e.g., date of birth, gender, address, and birthplace). Additionally, we cross-referenced our analysis with a recent CDC assessment that identified laws regarding collection of demographic data elements in IIS, including date of birth, address/zip code, sex/gender, citizenship/place of birth, and race/ethnicity.^1^

**Component 2. Vaccination Reporting Requirements**

*2b. Adult reporting requirements*

Example of reporting requirements for specific provider types.

Per Florida Law,

“[a] health care practitioner . . . who administers vaccinations . . . to college or university students from 18 years of age to 23 years of age at a college or university student health center or clinic is required to report vaccination data to the immunization registry . . .”^2^

*2c. Mechanisms to enforce reporting requirements*

Example of enforcement requirements for vaccine reporting.

Per North Dakota law,

“[i]f a health care provider fails to submit an immunization report required under this section within four weeks of vaccination: a. That health care provider may not order or receive any vaccine from the North Dakota immunization program until that provider submits all reports required under this section. b. The department of health and human services shall make a report to that health care provider's occupational licensing entity outlining that provider's failure to comply with the reporting requirements . . .”^3^

**Component 3. Patient consent required for data inclusion in IIS**

*3a.* *Example of consent for inclusion of pediatric data in IIS (often parent/guardian consent)*

Under Connecticut law,

“[t]he parent or guardian of any child who is listed or eligible to be listed in the immunization registry shall receive a written informational statement from the department about the immunization registry at the time of birth or, for qualifying children who come to Connecticut after birth, at the time of their coming to the attention of the immunization registry. Such statement shall inform the parent or guardian that their child's immunization information will be reported to and maintained by the immunization registry and that they may submit a written request to the immunization registry at any time requesting that their child's immunization record no longer be maintained. Once the request is received, the immunization registry shall no longer update nor make available that child's immunization record . . . ”^4^

Per Florida law,

“[a] health care practitioner . . . who administers vaccinations or causes vaccinations to be administered to college or university students from 18 years of age to 23 years of age at a college or university student health center or clinic is required to report vaccination data to the immunization registry, unless the student has refused to be included in the immunization registry by meeting the requirements of subparagraph 3. Vaccination data for students in other age ranges may be submitted to the immunization registry **only if the student consents to inclusion** in the immunization registry.”^2^

*3c. Consent for vital statistics data sharing with IIS*

The laws in 13 states and NYC (26%), dictated that it was mandatory, with no opt out, for vital statistics data, such as birth records (including vaccination data), to be shared with the IIS. In seven states (13%), patient consent is implied; however, patients or patients’ guardians could choose to opt out or to exclude the child’s or their data from the IIS. Lastly, 3 states (6%), KS, NH, and TX, required express consent for data to be shared between Vital Statistics and IIS. Under TX law, consent was defined to be express written consent, whereas NH law does not specify the format of consent, only that “indication from one parent” is needed.^5^ Per Texas law, consent is defined as

“. . .[a] statement signed by an individual or the individual's legally authorized representative agreeing that the individual's immunization history can be included in the immunization registry and that the individual's immunization record may be released from the immunization registry.”^6^

Lastly, the law in 29 states, DC, and PHI (55%) does not expressly require vaccination data-sharing between Vital Statistics or birth records and IIS. Data-sharing and reporting may still occur in these states; however, it is not expressly identified in the law.

**Component 4. Data sharing provisions (intrastate and interstate)**

*4a. Example of intrastate data-sharing permissions*

West Virginia law states that,

“[i]mmunization data that must be reported to the department is confidential, except it may be shared with other health care providers, or other entities with a legally defined access to the data, who are enrolled in the system, without the specific consent of the parent or patient. The data shall only be used for the ongoing care of the patient to assess immunization status, to determine immunization coverage rates, to assist in outbreak investigations or for other purposes determined by the Commissioner. . .

6.9. All of the data in WVSIIS is confidential and exempt from disclosure. In certain circumstances WVSIIS may release immunization information to the following:

6.9.a. A licensed physician, a licensed health care facility or other licensed healthcare provider in the state of West Virginia for the purpose of delivering medical or immunization services or for the purpose of identifying under-vaccinated persons;

6.9.b. A local health department for the purposes of delivering medical or immunization services or investigating or managing an outbreak or other reportable disease;

6.9.c. A school official for the purpose of determining if enrolled children have all of the immunizations required by W. Va. Code §§ 16-3-4 and the Bureau’s rule, Immunization Requirements and Recommendations for New School Enterers, 64CSR95, or for the prevention or control of vaccine-preventable disease within the school; and

6.9.d. Other appropriate persons for public health purposes and to prevent or control the spread of communicable disease.”^7^

Per New Mexico law,

“[a]ccess to the information in the immunization registry shall be limited to primary care physicians, nurses, pharmacists, managed care organizations, school nurses and other appropriate health care providers or public health entities . . .”^8^

*4c. Data-sharing requirements*

The requirements for intrastate and interstate data sharing vary across jurisdictions. For example, per IN law, “[b]efore immunization data may be released to a person or an entity, the person or entity must enter into a data use agreement with the state department that provides that information that identifies a patient will not be released to any other person or entity without the written consent of the patient unless the release is to a person or entity described in subsection (c).”^9^ Similarly, under AL law, “[b]y memoranda of understanding, the department may permit electronic access to the immunization registry via computer systems operated by other immunization data users.”^10^

Other states, such as WY, were more technically prescriptive with data sharing requirements. Under Wyoming law,

“(a) To achieve interoperability between the IIS and the electronic health record (EHR) system utilized by the organization or its facilities, a responsible authority or his designee for the organization shall:

(i) Submit a request for interoperability with the IIS using the form and process established by the Department; and

(ii) Identify an individual to serve as the project lead for each facility.

(b) The facility project lead shall:

(i) Identify a project team for the facility;

(ii) Demonstrate the EHR system's ability to comply with the Health Level Seven (HL7) protocol specifications established by the Department; and

(iii) Ensure compliance with the interoperability processes and procedures established by the Department.”^11^

**Component 5. School and childcare use of IIS**

*5c. Disclosure of vaccination records to schools and childcare facilities*

In 43 states, DC, and NYC (85%) IIS records can be directly disclosed to schools or childcare facilities for enrolled individuals. Wyoming law included an additional requirement to “ensure that qualifying parental consent has been obtained and documented . . . prior to the school administrator or the administrator's designee accessing a child's immunization record in the IIS. . .”^9^ Vermont law also required written parental consent for registry information to be released to childcare providers; however, consent was not required for schools. Laws in an additional four states provided for general vaccination information sharing with schools and childcare facilities through broader public health data sharing provisions but did not specify sharing from an IIS.

Per Idaho law,

“(3) The department of health and welfare shall only disclose information relating to an individual child in the registry to the following upon a specific request: (a) Employees of the health district in which the child resides or seeks medical services; (b) Health records staff of the school or school district in which the child is enrolled; (c) The operator of a licensed daycare facility in which the child is enrolled . . .“^12^

**Component 6. Inclusion of demographic data (eg, race/ethnicity)**

Example of minimum data elements to report.

Per Arkansas law,

“The minimum data elements to be furnished by the Provider in an immunization record in the immunization registry are: . . . Name, Date of Birth, Address, Mother's Name (Maiden name preferred), Gender & Race, . . . Insurance Status, All previous immunizations if not given by the reporting Provider . . .”^13^

**Exhibit A1**. Framework of Coding Questions and Possible Answers.

| Coding Question | Possible Coding Answer(s) |
| --- | --- |
| 1. What age groups are included in the IIS? | All ages  Children only (0-18)  Not mentioned |
| 1. Are any entities required to report pediatric vaccine administration to the IIS? | 0 – no  1 – yes  3 – unclear  Not mentioned |
| (2a) If yes, who? | All vaccine providers  Pharmacists only  Other – specify in comments  Not mentioned |
| 1. Are any entities required to report adult vaccine administration to the IIS? | 0 – no  1 – yes  3 – unclear  Not mentioned |
| (3a) If yes, who? | All vaccine providers  Pharmacists only  Medicaid/public/VFC providers only  Other – specify in comments  Not mentioned |
| 1. If any provider or entity is required to report vaccinations, is there a mechanism in place to enforce the requirement? | No requirement  Penalty – specify in comments  Resource-linked  Unclear – specify in comments  Not mentioned |
| 1. What type of consent is required from a parent before Vital Statistics shares birth record information with your IIS? | Expressed consent (verbal or written)  Expressed consent (written)  Implied consent (with opt out)  Mandated (no opt out)  Not mentioned  Unclear – specify in comments |
| 1. What type of consent is required from a parent before reporting vaccination information for their child to your IIS? | Expressed consent (verbal or written)  Expressed consent (written)  Implied consent (with opt out)  Mandated (no opt out)  Not mentioned  Unclear – specify in comments |
| 1. What type of consent is required from an adult before their vaccination information is reported to the IIS? | Expressed consent (verbal or written)  Expressed consent (written)  Implied consent (with opt out)  Mandated (no opt out)  N/A – adults not included in IIS  Not mentioned  Unclear – specify in comments |
| 1. Does the IIS law permit intrastate data sharing provisions? | 0 – no  1 – yes  3 – unclear  Not mentioned |
| (8a) If yes, what data-sharing requirements must be in place? | Free text |
| 1. Does the IIS law expressly permit interstate data sharing? | 0 – no  1 – yes  3 – unclear  Not mentioned |
| (9a) If yes, what data sharing requirements must be in place? | Free text |
| 1. Is IIS record/certificate expressly identified as documentary proof of vaccination for school/childcare entry? | 0 – no  1 – yes  3 – unclear  Not mentioned |
| 1. Do schools/childcare have authority to access IIS? | 0 – no  1 – yes  3 – unclear  Not mentioned |
| (11a) If yes, do schools/childcare expressly have edit/write access to IIS? | 0 – no  1 – yes  3 – unclear  Not mentioned |
| 1. Is demographic data expressly required by IIS law? | 0 – no  1 – yes  3 – unclear  Not mentioned |
| (12a) If yes, what elements of demographic data are required? | Free text |
| 1. Do patients have direct access to view their IIS records through an online portal or application? | 0 – no  1 – yes  3 – unclear  Not mentioned |

**Exhibit A2: Legal Citations**

1. Title 19A, Reporting Information to and Releasing Information from the Connecticut Immunization Registry and Tracking System, 19a-7h-1 (May 1, 2000).
2. The New Jersey Immunization Information System (NJIIS): Mandatory participation for health care providers, 8:57-3.16 (Apr 4, 2022).
3. California Business and Professions Code. Division 2, Healing Arts. Chapter 7, Optometry. Article 3, Admission to Practice: 3041(d) (Jan 1, 2022).
4. California Business and Professions Code. Chapter 9, Pharmacy, Section 4052.8 (Jan 1, 2022).
5. Illinois Register, Department of Public Health. Immunization Registry Code: Section 689.40 Immunization Data Provided to the Registry (Dec 13, 2021).
6. Illinois General Assembly. Professions, Occupations, and Business Operations: Illinois Dental Practice Act, 225 ILCS 25/54.3 (July 26, 2019).
7. Title 310, Oklahoma State Department of Health. Chapter 395. Licensed Midwives, 310:395-5-14 (Feb. 21, 2021).
8. 2020 Florida Statutes, Title XXIX Public Health. Chapter 381 Public Health: General Provisions, Section 003 Communicable disease and AIDS prevention and control (2020).
9. Rules and Regulations Pertaining to Immunization Reporting. The Arkansas State Board of Health. Section IV General Requirements, 007.15.10-IV (Nov 2015).
10. Oregon Health Authority. Public Health Division - Chapter 333. Division 49, Immunization Information Systems: 333-049-0050, Reporting to the Immunization Information System (IIS) (July 12, 2021).
11. Oregon Board of Dentistry. Chapter 818. Division 12, Standards of Practice: 818-012-0007 Procedures, Record Keeping and Reporting (Jan 1, 2020).
12. Code of Maine Rules. 10-144 Department of Human Services, Bureau of Health. Chapter 274: Immunization Information System (IIS) Rules: 11 Liability, Penalties, and Protections (Apr 16, 2002).
13. Oregon Health Authority. Public Health Division - Chapter 333. Division 49, Immunization Information Systems: 333-049-0030, Enrollment (Nov 12, 2018).
14. Annotated Code of Maryland. Health – General: 18-109, ImmuNet Program (Oct 1, 2019).
15. Missouri Legislature. Title XXII Occupations and Professions, Chapter 338. 338.010 Practice of Pharmacy Defined (Aug 28, 2021).
16. Montana Code Annotated 2021. Title 37, Professions and Occupations. Chapter 7, Pharmacy. Part 1, General. Administration of Immunizations, 37-7-105 (2021).
17. Utah Office of Administrative Rules. R386 Health, Disease Control and Prevention, Epidemiology. R386-800, Immunization Coordination. R386-800-3, Participation by Individuals (Sept 23, 2020).
18. The New Jersey Immunization Information System (NJIIS): Registrant Enrollment, 8:57-3.12 (Apr 4, 2022).
19. Title 64. Legislative Rule West Virginia Department of Health and Human Resources. 64-7-6 - Other Reportable Events: Administration of Immunizations. (Mar 18, 2022).
20. New Mexico Statutes. Chapter 24 - Health and Safety. Article 5 – Immunization. Section 24-5-9 - Access (Jun 17, 2005).
21. Department of Community Health. Community Public Health Agency. Michigan Care Improvement Registry. R 325.168, Exchange of Records (2009).
22. Code of Maine Rules. 10-144 Department of Human Services, Bureau of Health. Chapter 274: Immunization Information System (IIS) Rules: 10 Collection and Release of Information (Apr 16, 2002).
23. Commonwealth of Massachusetts. General Laws Part 1, Title XVI, Chapter 111, Section 24M: Computerized Immunization Registry (July 1, 2021).
24. Code of Colorado Regulations. Rule 6 CCR 1009-2 The Infant Immunization Program and Immunization of Students Attending School. Section 6 CCR 1009-2-VI – Official school immunization records (Jan 14, 2021).
25. Arkansas State Board of Health. Rules and Regulations Pertaining to Immunization Requirements, Promulgated Under the Authority of Ark. Code Ann. 20-7-109, 6-18-702, 6-60-501 – 504, and 20-78-206. Section III Immunization Requirements (Sept 1, 2014).
26. The New Jersey Immunization Information System (NJIIS): Authorized User Access to Information, 8:57-3.9 (Apr 4, 2022).
27. Oregon Health Authority. Public Health Division - Chapter 333. Division 49, Immunization Information Systems: 333-049-0070, Limitations on Access to Information in the Immunization Information System and Tracking and Recall System (Nov 12, 2018).
28. South Dakota Legislature. Administrative Rules, 44:81:03:02 Documentation Requirements (July 1, 2016).
29. Rules and Regulations Pertaining to Immunization Reporting. The Arkansas State Board of Health. Section IV General Requirements, 007.15.10-III (Nov 2015).
30. Hawaii State Legislature. §461-11.4 Vaccinations; children (2012).

**References**

1. Centers for Disease Control and Prevention. State Immunization Information System Laws – Demographic Data Collection [Internet]. Atlanta (GA): CDC; 2022 [cited 2022 Dec 19]. Available from: https://www.cdc.gov/phlp/docs/IIS_Sociodemo.pdf
2. 2020 Florida Statutes, Title XXIX Public Health. Chapter 381 Public Health: General Provisions, Section 003 Communicable disease and AIDS prevention and control (2020).
3. North Dakota Legislative Branch. North Dakota Century Code. Title 23, Chapter 22-01: State Department of Health. 23-01-05.3 Immunization Data (Sept 23, 2022).
4. Title 19A, Reporting Information to and Releasing Information from the Connecticut Immunization Registry and Tracking System. 19a-7h-5, Refusing participation in the immunization registry (May 1, 2000).
5. Chapter 5-C New Hampshire Vital Records Administration (NHVRIN). Birth Registration Forms and Procedures: Section 5-C:19 (Jan 1, 2006).
6. Texas Administrative Code. Title 25: Health Services. Part 1: Department of State Health Services. Chapter 100: Immunization Registry. Rule § 100.1: Definitions (May 6, 2004).
7. Title 64. Legislative Rule West Virginia Department of Health and Human Resources. 64-7-6 - Other Reportable Events: Administration of Immunizations. (Mar 18, 2022).
8. New Mexico Statutes. Chapter 24 - Health and Safety. Article 5 – Immunization. Section 24-5-9 - Access (Jun 17, 2005).
9. Indiana Code 16-38-5. Chapter 5, Immunization Data Registry. IC 16-38-5-3 (2016).
10. Rules of the State Board of Health, Bureau of Communicable Disease. Chapter 420-6-2 – Exchange of Immunization Information and Operation of the Alabama Immunization Registry. 420-6-2-.04, Access to the Immunization Registry (Dec 5, 1997).
11. Wyoming Secretary of State. Health, Department of (048): Wyoming Immunization Program (0071). Chapter 5: Wyoming Immunization Information System, Section 10 (Feb 7, 2018).
12. Idaho Statutes. Title 39, Health and Safety. Chapter 48, Immunization. 39-4803, Immunization Registry (July 1, 2022).
13. Arkansas State Board of Health. Rules and Regulations Pertaining to Immunization Requirements, Promulgated Under the Authority of Ark. Code Ann. 20-7-109, 6-18-702, 6-60-501 – 504, and 20-78-206. Section III Immunization Requirements (Sept 1, 2014).
